# Alaskan brown bears (*Ursus arctos*) aggregate and display fidelity to foraging neighborhoods while preying on Pacific salmon along small streams

**DOI:** 10.1002/ece3.4431

**Published:** 2018-08-19

**Authors:** Aaron J. Wirsing, Thomas P. Quinn, Curry J. Cunningham, Jennifer R. Adams, Apryle D. Craig, Lisette P. Waits

**Affiliations:** ^1^ School of Environmental and Forest Sciences University of Washington Seattle Washington; ^2^ School of Aquatic and Fishery Sciences University of Washington Seattle Washington; ^3^ Department of Fish and Wildlife Sciences University of Idaho Moscow Idaho

**Keywords:** genetic capture‐mark‐recapture, noninvasive population estimation, *Oncorhynchus nerka*, predation, sockeye salmon

## Abstract

The interaction between brown bears (*Ursus arctos*) and Pacific salmon (*Oncorhynchus* spp.) is important to the population dynamics of both species and a celebrated example of consumer‐mediated nutrient transport. Yet, much of the site‐specific information we have about the bears in this relationship comes from observations at a few highly visible but unrepresentative locations and a small number of radio‐telemetry studies. Consequently, our understanding of brown bear abundance and behavior at more cryptic locations where they commonly feed on salmon, including small spawning streams, remains limited. We employed a noninvasive genetic approach (barbed wire hair snares) over four summers (2012–2015) to document patterns of brown bear abundance and movement among six spawning streams for sockeye salmon, *O. nerka*, in southwestern Alaska. The streams were grouped into two trios on opposite sides of Lake Aleknagik. Thus, we predicted that most bears would forage within only one trio during the spawning season because of the energetic costs associated with swimming between them or traveling around the lake and show fidelity to particular trios across years because of the benefits of familiarity with local salmon dynamics and stream characteristics. Huggins closed‐capture models based on encounter histories from genotyped hair samples revealed that as many as 41 individuals visited single streams during the annual 6‐week sampling season. Bears also moved freely among trios of streams but rarely moved between these putative foraging neighborhoods, either during or between years. By implication, even small salmon spawning streams can serve as important resources for brown bears, and consistent use of stream neighborhoods by certain bears may play an important role in spatially structuring coastal bear populations. Our findings also underscore the efficacy of noninvasive hair snagging and genetic analysis for examining bear abundance and movements at relatively fine spatial and temporal scales.

## 1 INTRODUCTION

Each year, hundreds of millions of adult Pacific salmon (*Oncorhynchus* spp.) transport vast quantities of energy and marine‐derived nutrients to freshwater ecosystems in Asia and North America when they return from the ocean to spawn in streams, rivers, and lakes (Gresh, Lichatowich, & Schoonmaker, 2000; Hocking & Reynolds, 2011; Schindler, Scheuerell, et al., 2003). These salmon subsidies, in turn, are exploited by a variety of freshwater and terrestrial consumers (e.g., bears, Hilderbrand, Schwartz, et al., 1999; birds, Field & Reynolds, 2011; fishes, Bilby, Fransen, & Bisson, 1996; Scheuerell, Moore, Schindler, & Harvey, 2007; invertebrates, Bilby et al., 1996; Winder, Schindler, Moore, Johnson, & Palen, 2005) and can even alter nutrient cycling in riparian soils (Holtgrieve, Schindler, & Jewett, 2009) and affect both the productivity and structure of associated plant communities (Helfield & Naiman, 2001; Hocking & Reynolds, 2011). Consequently, the anadromous life cycle of Pacific salmon has become a celebrated example of animal movement coupling distinct ecosystems. Yet, the effects of the salmon influx on freshwater and riparian ecosystem processes are heterogeneous and context dependent (Hocking & Reimchen, 2009; Hocking & Reynolds, 2011; Holtgrieve et al., 2009). Accordingly, studies are needed to better understand factors mediating spatiotemporal variation in the impacts of salmon anadromy on both the aquatic and terrestrial food webs that are associated with their spawning habitat (Schindler, Scheuerell, et al., 2003).

Bears (brown *Ursus arctos*; and black *U. americanus*) can play a pivotal role in mediating the flow of salmon energy and nutrients into freshwater and riparian ecosystems. Bears often drag carcasses, deposit unconsumed flesh, and excrete consumed salmon far from the place of capture; thus, their foraging activity helps to determine the extent to which salmon nutrients enter aquatic versus terrestrial ecosystem pathways (Helfield & Naiman, 2006; Hilderbrand, Hanley, Robbins, & Schwartz, 1999; Hocking & Reimchen, 2009; Meehan, Seminet‐Reneau, & Quinn, 2005). For example, Helfield and Naiman (2006) found that the influx of nitrogen to riparian forests in southwestern Alaska was greatly enhanced in the presence of both brown bears (*U. arctos*) and Pacific salmon compared to areas occupied by either species independently. The degree to which bears act as vectors for salmon biomass, however, is spatially and temporally variable, hinging on such factors as watershed characteristics that influence salmon accessibility (e.g., stream width and depth) as well as both salmon and bear density (Hilderbrand, Hanley, et al., 1999; Quinn, Carlson, Gende, & Rich, 2009; Quinn, Cunningham, & Wirsing, 2017). Whereas previous studies have quantified the influence of stream traits and salmon density on carcass deposition by bears (e.g., Quinn et al., 2009), the difficulty associated with estimating bear abundance at relevant spatial and temporal scales has hindered assessment of the relationship between bear density and the delivery of salmon‐derived nutrients to terrestrial food webs. This knowledge gap is especially pronounced in areas where small salmon spawning streams predominate, which typify the habitats along the north Pacific Rim where bears and salmon interface but challenge bear observation and enumeration (Quinn, Wirsing, Smith, Cunningham, & Ching, 2014). In contrast, bear–salmon interactions are more easily observed at the few less typical places where large numbers of bears congregate to feed on salmon passing through a migratory bottleneck, such as the McNeil River, Alaska (Gill & Helfield, 2012; Sellers & Aumiller, 1994). Thus, studies that reveal patterns of bear abundance and foraging behavior along small spawning streams at ecologically relevant spatial and temporal scales are crucial to understanding the processes by which salmon nutrients and biomass are transported across aquatic‐terrestrial boundaries in more representative habitats.

**Figure 1:**
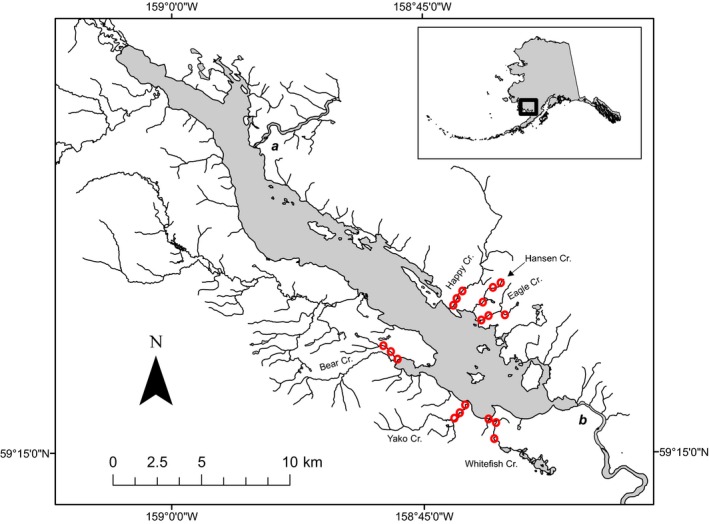
Map showing the location of the study area in Alaska (black box in the inset), Lake Aleknagik (gray), and the six streams where bears were studied using hair sampling (Happy, Hansen, Eagle, Bear, Yako and Whitefish creeks). Bears traveling around the lake would need to ford either the Agulowak River to the north (a) or the Wood River to the south (b). Barbed wire placements are indicated by the red circles; two wires per stream were deployed at any given time [Colour figure can be viewed at http://wileyonlinelibrary.com]

Along the shores of Lake Aleknagik, in southwestern Alaska, sockeye salmon (*Oncorhynchus nerka*) are available as prey for brown bears as the salmon ascend and spawn in a series of small streams (Quinn, Gende, Ruggerone, & Rogers, 2003; Quinn, Wetzel, Bishop, Overberg, & Rogers, 2001). Each summer for more than two decades, salmon falling prey to bears in many of these streams have been recorded systematically using surveys of live and dead fish, yielding a detailed understanding of the relationship between bear predation rates and spatiotemporal variation in salmon abundance (Quinn et al., 2017). Yet, until recently, the numbers and movements of bears foraging on these streams have remained a mystery, owing in large part to the difficulty of observing and discriminating between individuals without disturbing their interactions with salmon. In 2012, however, we began a noninvasive approach—unbaited hair snares—to sample individual bears along six streams grouped into two trios (or “neighborhoods,” see below) on either side of the lake (Figure 1). Then, during the summers of 2013–2015, we used hair samples collected with these snares as the basis for generating noninvasive genetic capture–mark–recapture (CMR) estimates of the number of females and males using each stream. The results of this CMR analysis allowed us to characterize not only spatiotemporal patterns of bear abundance in our system but also site fidelity and factors influencing differences in detection rates.

There is growing evidence that brown bears exhibit optimal foraging strategies for salmon both within streams (Cunningham, Ruggerone, & Quinn, 2013; Gende, Quinn, Hilborn, Hendry, & Dickerson, 2004; Klinka & Reimchen, 2002) and across large landscapes (Deacy, Leacock, Armstrong, & Stanford, 2016). Accordingly, under the hypothesis that individual bears have knowledge of foraging opportunities across the landscape and respond to differences in salmon densities in a manner that optimizes energy intake rate, we predicted that most individuals would: (a) use only one stream neighborhood during a spawning season (summer) because of the energetic costs associated with swimming across or traveling around the lake; and (b) show fidelity to stream neighborhoods across years because of the presumed benefits of familiarity with local salmon dynamics (e.g., run timing) and stream characteristics (e.g., prime fishing areas) (Edwards, Nagy, & Derocher, 2009; Part, 1995). Bears are highly mobile consumers (Rode, Farley, Fortin, & Robbins, 2007) and the salmon streams in our system are in relatively close proximity (~1–7 km; Figure 1), so we expected to observe frequent movement between streams within neighborhoods in a given year. However, average annual salmon run size varies among the streams comprising both stream neighborhoods (Quinn et al., 2017), so we did not expect stream usage patterns to be uniform. Rather, because we expect individual bears to spend more time foraging along streams with larger salmon runs (Hilderbrand, Hanley, et al., 1999) and that residency influences probability of detection, we further predicted that (c) detection rates would vary among streams, even after accounting for neighborhood fidelity, with relatively productive (i.e., perennially salmon rich) streams exhibiting the highest detection rates. Bear activity is affected by human presence, and some bears are more responsive than others (Martin et al., 2010; Olson, Gilbert, & Squibb, 1997; Ordiz, Støen, Delibes, & Swenson, 2011; Rode, Farley, & Robbins, 2006a). In particular, females tend to be less sensitive to human presence than males, at least in part because females with cubs appear to use humans as shields against male‐induced infanticide (Rode, Farley, & Robbins, 2006b; Steyaert, Kindberg, Swenson, & Zedrosser, 2013; Steyaert et al., 2016). Because humans occasionally visit our focal streams for research and bears without cubs in the region are hunted, we also predicted that (d) females would be more abundant and detected more often than males owing to sex‐specific differences in human avoidance behavior.

## 2 MATERIALS AND METHODS

### 2.1 Study sites

We sampled six streams flowing into Lake Aleknagik in the Wood River system of southwestern Alaska that are similar in size (all ≤6.4 km long with most salmon spawning in the lower 2–3 km) and proximal to each other (see Quinn et al., 2014 for details; Figure 1). All of our study streams are used for spawning by sockeye salmon, feature predictable patterns in salmon spawning timing and abundance, and experience well‐documented predation by bears during annual salmon runs (Quinn et al., 2001, 2003, 2014, 2017). Moreover, spawning by other Pacific salmon species has only rarely been observed in any of these streams (Pess, Quinn, Schindler, & Liermann, 2014), facilitating analysis of bear abundance and behavior in relation to sockeye salmon without any confounding effects of other salmon species as prey. Three streams flow into each side of the portion of Lake Aleknagik included in our study: Happy, Hansen, and Eagle creeks are on the northeast side of the lake, and Bear, Yako, and Whitefish creeks are on the southwest side. The width of the lake separating these two stream trios is approximately 4 km, so we considered it possible but unlikely that bears would move directly (by swimming) from one set to the other within the spawning season. The alternative route would be for a bear to travel south from either Eagle Creek or Whitefish Creek, swim across the Wood River (ca. 70–100 m wide) and then travel north along the other shore, a considerably longer distance but primarily by land. Finally, it would be possible to travel the much longer distance north (uplake to the northwest), across the Agulowak River, and down the other shore (Figure 1). In contrast to the considerable travel distances necessary for these alternatives, short distances between stream mouths within each stream trio (average = 2.0 km) and the absence of any geographic barriers facilitated interstream movement. Accordingly, we designated each stream trio as a putative bear foraging neighborhood (i.e., an area likely to fall within the zone of influence of individual bears during a spawning season; sensu Addicott et al., 1987) and refer to them simply as “neighborhoods.” Other than periodic surveys by researchers, human presence on these streams is minimal because they are too small to support recreational fisheries and there is no human habitation beyond the lake shore. However, bears in the wider region surrounding Lake Aleknagik are subject to hunting in spring and fall, as regulations permit.

### 2.2 Bear hair sampling

**Figure 2:**
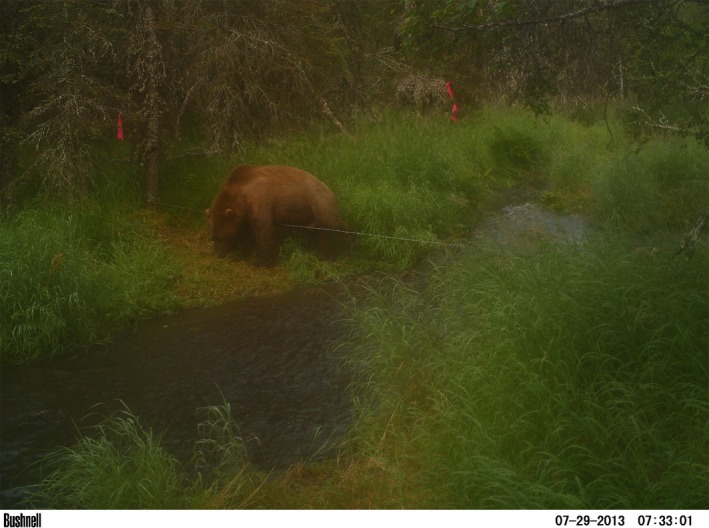
Bear hair sampling wires were deployed in the first 2 km of six streams flowing into Lake Aleknagik (Wood River System, AK) over the course of four summers (2012–2015). Here, a brown bear captured by a motion‐activated trail camera steps over a barbed wire strand deployed on Hansen Creek [Colour figure can be viewed at http://wileyonlinelibrary.com]

Bear hair sampling for DNA analysis and individual identification is typically undertaken using barbed wire surrounding bait stations (Shardlow & Hyatt, 2013; Woods et al., 1999), but samples have also been collected with unbaited snares deployed in areas regularly travelled by bears (Beier, Lewis, Flynn, Pendleton, & Schumacher, 2005; Haroldson et al., 2005; Robinson, Waits, & Martin, 2009). Accordingly, with the intention of sampling hair from brown bears transiting through the stream corridor, we deployed one strand of unbaited four‐pronged barbed wire with a 12‐cm barb interval at two locations on each of three streams (Bear, Happy, and Hansen) in 2012 (Quinn et al., 2014). The wires were strung directly across the stream and anchored to trees on either side using fencing staples (Figure 2). Wires were tightened to parallel the stream's surface at a height of 50–55 cm above the streambed at mid‐channel, which is the recommended height for snagging hair from bears stepping over or underneath the wire (Haroldson et al., 2005; Long, MacKay, Zielinski, & Ray, 2008). Only one strand was deployed per sampling site, rather than adding a lower wire to sample cubs, given that single wires sample the majority of passing bears, including family groups (Boulanger et al., 2006). During the summers of 2013–2015, we expanded wire sampling to all six streams, again deploying two wires per stream. In all years, we only sampled the first 2 km of each stream (starting from the mouth) and wire length averaged 8 m. In all years, wire locations on each stream were moved periodically to minimize the possibility of habituation (Boulanger et al., 2006).

Each year, wires were checked every other day to limit hair sample degradation (Piggott, 2004; Taberlet, Waits, & Luikart, 1999; Waits & Paetkau, 2005). In 2012, we sampled hair for a short duration as a pilot effort, whereas sampling in the following 3 years spanned the salmon spawning season in these streams. Specifically, we collected hair from the wires from 20 July to 29 July in 2012, 14 July to 18 August in 2013, 19 July to 26 August in 2014, and 19 July to 27 August in 2015. Wires were scanned for the presence of hair, and any hair present was collected with sterilized (burned) forceps and placed in a coin envelope. Any location on the wire that had snagged hair was sterilized using a butane torch following sample collection, and care was also taken to avoid direct contact between hair samples and the researchers collecting them to avoid contamination (Waits & Paetkau, 2005). We noted the location of each hair sample on the wire by counting barb numbers to facilitate discrimination of samples likely to have been left simultaneously by the same bear from those potentially deposited by multiple bears. Collected samples were stored initially in silica desiccant and later frozen prior to being analyzed at the Laboratory for Ecological, Evolutionary, and Conservation Genetics at the University of Idaho.

### 2.3 Laboratory analysis

Genetic analysis of hair samples is expensive, so we employed a subsampling approach designed to avoid extracting redundant samples left by the same bear following an encounter with a wire while maximizing the likelihood of obtaining a sample from as many different bears as possible (Long et al., 2008; Tredick, Vaughan, Stauffer, Simek, & Eason, 2007). Specifically, we analyzed (a) any hair sample that was the only sample found on a wire on a given day; and (b) only the highest quality sample (i.e., the sample with the greatest number of hair follicles; Tredick et al., 2007) in cases where samples were found on a series of adjacent barbs on a particular wire checked on a given day, under the assumption that these “sample clusters” were deposited by the same bear (Long et al., 2008). This second sampling rule resulted in multiple samples being analyzed from a particular wire checked on a given day, if >1 hair sample cluster was discovered (e.g., on opposite ends of the wire). Bears could have been present in the area but never encountered the wires, so the population estimates are necessarily conservative. However, visual evidence from motion‐activated game cameras and our observations of bear trails through the riparian underbrush suggest the bears in our study system often transit along salmon streams or on banks rather than through the surrounding forest.

DNA was extracted from each hair sample selected for analysis using the DNeasy Blood and Tissue Kit (Qiagen, Inc.). Based on visual observations and camera data, all samples were assumed to be brown bears. This assumption was confirmed by PCA analysis in Genalex (Peakall & Smouse, 2006), and no outlier samples were detected. A genotype was generated for each sample using 10 nuclear DNA microsatellite loci (Bellemain & Taberlet, 2004; De Barba et al., 2010; Paetkau, Shields, & Strobeck, 1998; Paetkau & Strobeck, 1994; Taberlet et al., 1997) and one sex identification marker (Ennis & Gallagher, 1994). The polymerase chain reaction (PCR) contained 0.03 μM of G10C, 0.04 μM of SEY, 0.07 μM of G1D, Mu15, Mu23 and G1A, 0.10 μM of Mu50, 0.13 μM of G10P, 0.14 μM of G10M, 0.19 μM of G10H, 1× Qiagen Multiplex PCR Kit Master Mix, 0.5× Q solution and 2 μl of DNA extract in a 7‐μl reaction volume. The PCR thermal profile was an initial denaturation step of 94°C for 15 min followed by 20 cycles of 94°C for 30 s, 60°C touchdown −0.5°C per cycle for 90 s and 72°C for 60 s followed by 30 cycles of 94°C for 30 s, 50°C for 90 s, 72°C for 60 s with a final extension of 60°C for 30 min. Each sample was amplified two to four times to minimize and detect genotyping errors. Consensus genotypes were obtained following the rule that each allele needed to be observed twice at each locus, and a sample had to contain a consensus genotype at eight or more loci to be included in the matching analysis. Two genotypes were considered a match in the program Genalex (Peakall & Smouse, 2006) if they were identical, or if a one allele mismatch at two or fewer loci could be due to allelic dropout. The probability of a match for unrelated individuals across 10 loci was 0.0000000011, and the probability of a match between siblings across 10 loci was 0.00025 (Waits, Luikart, & Taberlet, 2001). For all single captures (individuals detected once), the reliability of their genotype was estimated using the program Reliotype (Miller, Joyce, & Waits, 2002) and retained in the dataset if the reliability score was ≥90%.

### 2.4 Statistical analysis

In 2012, we genetically identified all bears detected on the three streams that were sampled (Bear, Happy, and Hansen), but did not generate population estimates because of the limited sampling period. For each of the subsequent 3 years (2013–2015), we generated noninvasive genetic CMR estimates of the number of bears on our focal streams with a closed‐capture, multisession modeling approach in program MARK (White & Burnham, 1999) using the package RMark (Laake, 2013) in R (R Core Team, 2015). To do so, we first divided each of our full sampling seasons into three roughly 2‐week occasions and then built binary encounter histories for each individual bear detected (i.e., whose hair sample was successfully amplified) on each stream over the course of a season, with “1” indicating a detection and “0” indicating a nondetection during any particular occasion. Accordingly, only a single detection per individual was counted per 2‐week sampling occasion.

We used encounter histories to build Huggins closed‐capture models of bear abundance that incorporated factors potentially responsible for varying detection (or capture) probability (*p*) (Huggins, 1989; Kendall, Nichols, & Hines, 1997). These models assume no births, deaths, immigration, and emigration during the sampling period (White, Anderson, Burnham, & Otis, 1982). The relatively short duration of our sampling seasons (no more than 6 weeks) and the absence of any hunting during the times in which we sampled bear hair helped to minimize violation of the closed population assumption underpinning our approach. Furthermore, Kendall (1999) found that closed‐capture estimators are relatively unbiased when capture probability varies primarily with time, as in our case. This modeling approach allowed us to estimate bear numbers on each stream in each year (except 2012) while also quantifying the extent to which model fit was improved by allowing detection heterogeneity to vary as a function of one or more covariates that we expected might be important. These covariates included an estimable parameter for the effect of sampling occasion (2‐week interval) on detection probability, to account for the possibility that detection rates might change over the course of the summer. We also allowed detection probability to vary across streams to test our prediction that stream‐specific detection rates might differ. Furthermore, we estimated detection probability and abundance for females and males separately to test our prediction that stream use patterns would differ between the sexes. On any stream in a given year, we deemed abundance estimates for females and males with nonoverlapping 95% confidence intervals to indicate significant differences in use by the two sexes (Johnson, 1999). In the absence of bait and with the use of a noninvasive sampling technique, we considered it unlikely that the initial detection would influence the likelihood of subsequent detections. Thus, all models in each year's candidate set assumed equal detection and redetection probability (i.e., no aversion). For each year, we used Akaike's information criterion corrected for small sample size (AIC_c_) to evaluate relative support for candidate models of bear abundance; as part of this process, we visually inspected parameter estimates and standard errors in order to identify models within each candidate set that failed to run successfully (O'Brien & Kinnaird, 2011). We generated coefficient estimates and 95% confidence intervals for all parameters included in the top models for each year's candidate set (Burnham & Anderson, 2002). We also quantified the formal strength of evidence for the top model relative to the null model in each year using evidence ratios (Burnham, Anderson, & Huyvaert, 2011).

## 3 RESULTS

### 3.1 Sample collection and genetic analysis

Over the course of four summers we collected a total of 2,026 bear hair samples and then applied our subsampling approach to select 829 for genetic analysis (Supporting Information Table S1). We successfully amplified 524 samples (Table 1), yielding an overall success rate of 63% and annual success rates of 68% for 2012, 58% for 2013, 65% for 2014, and 65% for 2015.

**Table 1:** Numbers of detections (i.e., successful genotyping; detected) and unique individual brown bears (*Ursus arctos*) identified (IDs) using hair sampling barbed wires deployed along six sockeye salmon (*Oncorhynchus nerka*) spawning streams flowing into Lake Aleknagik (Wood River System, Alaska) over the course of four summers (2012–2015)

Stream	2012			2013			2014			2015		
	Detected	IDs	Salmon	Detected	IDs	Salmon	Detected	IDs	Salmon	Detected	IDs	Salmon
Happy (N)	6	4F/1M	6,285	34	9F/3M	1,953	5	2F/0M	27,559	39	10F/6M	11,104
Hansen (N)	12	7F/1M	4,228	55	11F/8M	4,410	15	5F/4M	55,663	55	14F/8M	8,286
Eagle (N)	–	–	251	20	4F/5M	1,243	38	12F/4M	6,261	46	11F/13M	1,443
Bear (S)	10	2F/2M	960	12	4F/1M	2,743	19	9F/2M	6,222	54	9F/5M	3,090
Yako (S)	–	–	691	24	8F/5M	1,172	15	6F/2M	13,081	40	8F/2M	2,246
Whitefish (S)	–	–	136	8	3F/3M	789	9	4F/1M	4,392	8	2F/3M	345

*Note*

Individuals are identified as females (F) or males (M). Streams are designated as comprising either the trio along the northern (N) or southern (S) shore of the lake. Salmon estimates are combined live and dead counts at the peak of abundance for each stream.

### 3.2 Detections

Our 524 detections yielded 121 individual brown bears (68 females, 51 males, and two individuals for which sex was not determined). No black bears or other species were detected. The number of individuals detected on a stream in a given year ranged from 2 to 24 (mean = 10.6), with as many as 14 females and 13 males detected on any one stream in a given year (Table 1). The number of detections (i.e., samples) per individual in a year ranged from 1 to 26, with single detections within a year comprising 56.25% of the total in 2012, 40.91% of the total in 2013, 52.50% of the total in 2014, and 36.51% of the total in 2015. The annual number of detections per individual averaged 3.83 (±4.47 *SD*) for females and 2.50 (±2.76 *SD*) for males.

### 3.3 Stream neighborhood use

During the 3 years in which all six streams were sampled, 16 of 44 individuals (36.4%) were detected along more than one stream within a neighborhood in 2013 (11 of 26 females; 42.3%; five of 18 males, 27.8%), 10 of 39 individuals (25.6%) were detected along more than one stream within a neighborhood in 2014 (seven of 27 females; 25.9%; three of 12 males, 25.0%), and 26 of 62 individuals (41.9%) were detected along more than one stream within a neighborhood in 2015 (17 of 34 females; 50.0%; nine of 28 males, 32.1%). Individuals using all three streams within a neighborhood constituted 6.8% of the detected cohort in 2013 (one of 26 females, 3.9%; two of 18 males, 11.1%), 5.13% in 2014 (two of 27 females, 7.4%; 0 of 12 males), and 6.5% in 2015 (three of 34 females, 8.8%; 1 of 28 males, 3.6%). Movement between stream neighborhoods within a year was rare. Specifically, only one female switched neighborhoods during the summer of 2014: we detected this bear 12 times from July 18 to 30 in the southern stream trio (Bear, Yako, Whitefish) and then 7 times from 10–22 August in the northern trio (Happy, Hansen, Eagle). We detected a total of 42 individuals in more than 1 year; numbers of recaptured females (30) and males (12) did not differ significantly from expectations based on the overall sex ratio of detected individuals (*χ* ^2^ = 3.567, *p *=* *0.059). Among these recaptures, nine females (30.0%) and two males (16.7%) were sampled in 3 years; this distribution was not significantly different from the one expected based on the overall sex ratio of detected individuals (*χ* ^2^ = 2.764, *p *=* *0.096). No individual was detected in all 4 years of the investigation, although one female and two males were sampled in the first and last years. Interannual movement between the stream trios was infrequent. Specifically, three individuals (7.14%) switched stream neighborhoods between years (one female, 3.3%; two males, 16.7%).

### 3.4 Abundance estimation

**Figure 3:**
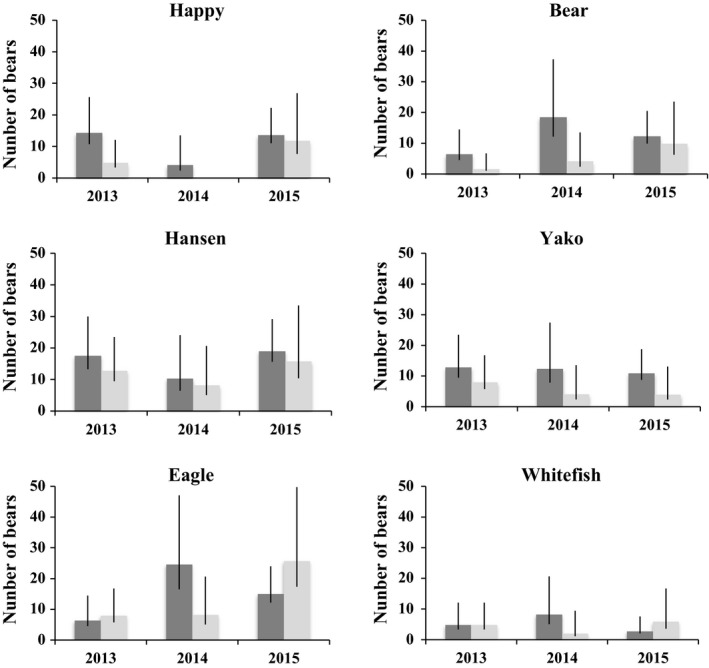
Noninvasive genetic capture–mark–recapture estimates of brown bear abundance along six sockeye salmon spawning streams that flow into Lake Aleknagik in Bristol Bay, Alaska. Estimates were generated using multi‐session Huggins closed‐capture models based on encounter histories for each stream over the course of three summers (2013–2015) during which hair samples were collected with barbed wire for 6 weeks. In each summer, the 6‐week sampling session was split into three, 2‐week occasions for modeling purposes. The dark gray columns represent estimates for females, and light gray columns represent estimates for males; the black lines depict 95% confidence intervals. The streams are grouped by columns into trios on the north (N; Happy, Hansen, Eagle) and south (S; Bear, Yako, Whitefish) sides of the lake

Our genetic CMR analysis focused on bears detected during the 3 years when all six streams were sampled and therefore excluded nine individuals (five females, four males) that were detected only in 2012. Overall, the models produced estimates indicating considerable and variable use of the six streams by bears within and across years (Figure 3, Supporting Information Table S2). In 2013, after rounding, model estimates ranged from eight individuals (six females, two males) on Bear Creek to 31 individuals on Hansen Creek (18 females, 13 males; Figure 3, Supporting Information Table S2). In 2014, estimates spanned four individuals on Happy Creek (four females, 0 males) to 33 individuals on Eagle Creek (25 females, eight males). In 2015, estimates ranged from nine individuals on Whitefish Creek (three females, six males) to 41 individuals on Eagle Creek (15 females, 26 males). Using these values, the estimated numbers of different bears on the streams averaged 29.23 on Eagle, 27.84 on Hansen, 17.52 on Bear, 17.27 on Yako, 16.21 on Happy, and 9.48 on Whitefish.

Female abundance estimates were higher than those for males in all years for four streams: Happy, Hansen, Bear, and Yako (Figure 3, Supporting Information Table S2). Conversely, male abundance was estimated to be higher in two of the 3 years on Eagle, whereas on Whitefish female and male abundance was estimated to be equivalent in 2013, female abundance was higher in 2014, and male abundance was higher in 2015 (Figure 3, Supporting Information Table S2). In all comparisons, however, overlapping 95% confidence intervals indicated that the estimated differences were not significant.

### 3.5 Patterns of detection

In 2013, bear detectability varied with time (sampling occasion). Namely the top model of bear abundance included time‐varying capture probability (*p*) (Table 2). The ∆AIC_c_ between the top and null (second‐place) models was 1.727 (Table 2); the evidence ratio between these two models suggested that the evidence for the top model was 2.371 stronger than that for the null. The coefficient estimate for the third occasion (−0.761, 95% CI = −1.403, −0.120) indicated a drop in detection probability relative to the reference state (occasion one) (Table 3). However, the estimated detection probabilities for occasions one (0.343) and three (0.196) had overlapping 95% CI (Table 3).

**Table 2:** Huggins multi‐session, closed‐capture models estimating brown bear abundance over the course of three summers (2013, 2014, 2015) along six salmon spawning streams flowing into Lake Aleknagik, AK

	Model	ΔAIC_c_	AIC_c_ weights	*K*	Deviance
2013	*p* (time)	0	0.585	3	227.238
	Null	1.727	0.247	1	233.071
2014	*p* (time)	0	0.801	2	159.434
	Null	2.783	0.199	1	166.352
2015	*p* (sex)	0	0.618	2	339.102
	Null	1.457	0.298	1	342.588

*Note*

Models comprising candidate sets for each year were ranked using Akaike information criteria corrected for small sample size (AIC_c_); only those models falling within ΔAIC_c_ ≤ 2 of the top model for a given year are presented, plus the null model. For each model, *K* represents the number of estimable parameters including the intercept; parameters available for inclusion in the models were variation in capture probability (*p*) as a function of *sex* (female vs. male, with female serving as the reference setting), *stream* (six streams with Happy serving as the reference setting), and *time* (2‐week sampling occasions with occasion one serving as the reference setting).

**Table 3:** Coefficient estimates (*β*) and detection probabilities (*p*) associated with parameters included in the top Huggins model of brown bear abundance along six salmon spawning streams flowing into Lake Aleknagik, AK for each of 3 years (2013–2015)

	Parameter	*β*	95% CI	*p*	95% CI
2013	Intercept	−0.651	−**1.236,** −**0.066**	—	—
	*p* (time, interval 1)	—	—	0.343	0.225, 0.483
	*p* (time, interval 2)	−0.226	−0.817, 0.365	0.294	0.189, 0.426
	*p* (time, interval 3)	−0.761	−**1.403,** −**0.120**	0.196	0.118, 0.307
2014	Intercept	−1.866	−**2.622,** −**1.110**	—	—
	*p* (time, interval 1)	—	—	0.134	0.068, 0.248
	*p* (time, interval 2)	0.362	−0.389, 1.114	0.182	0.097, 0.316
	*p* (time, interval 3)	0.910	**0.195, 1.624**	0.278	0.155, 0.446
2015	Intercept	−0.577	−**1.013,** −**0.142**	—	—
	Female	—	—	0.360	0.266, 0.465
	Male	−0.745	−1.558, 0.067	0.210	0.118, 0.346

*Note*

95% confidence intervals (CI) are given for each coefficient and detection probability estimate; those for *β* that do not overlap zero are bolded. Note that, for each year, time interval (i.e., occasion) one and female served as the reference setting.

As in 2013, bear detectability varied with time in 2014. That is, the top model included time‐varying capture probability, and the ∆AIC_c_ between this model and the runner‐up (null) was 2.783 (Table 2). The evidence ratio indicated that the strength of evidence for the top model was 4.021 greater than that for the null. The coefficient estimate for the third occasion (0.910, 95% CI = 0.195, 1.624) indicated an increase in detection probability relative to the reference state (occasion one), but the estimated detection probabilities for occasions one (0.134) and three (0.276) had overlapping 95% CI (Table 3).

In 2015, bear detectability varied between the sexes. Namely the top model included variation in capture probability as a function of sex, and the ∆AIC_c_ between this model and the runner‐up (null) was 1.457 (Table 2). The evidence ratio indicated that support for the top model was 2.072 greater than that for the null model. The coefficient estimate for males (−0.745) suggested reduced detection probability relative to females, but the 95% CI for this estimate overlapped zero (−1.558, 0.067; Table 3). Furthermore, the estimated detection probabilities for females (0.360) and males (0.210) had overlapping 95% CI (Table 3).

## 4 DISCUSSION

The ecological importance of the predator‐prey relationship between brown bears and Pacific salmon is widely recognized (Helfield & Naiman, 2006; Hilderbrand, Hanley, et al., 1999; Hilderbrand, Schwartz et al., 1999). Yet, much of what we know about the numbers of bears occupying specific locations where this interaction occurs comes from either a few exposed areas along the Brooks and McNeil rivers in Alaska where bears are relatively easy to observe (e.g., Gill & Helfield, 2012; Olson et al., 1997) or studies relying on abundance proxies such as camera trapping detections (e.g., Levi, Wheat, Allen, & Wilmers, 2015; Quinn et al., 2014; Schindler et al., 2013). Consequently, our understanding of the localized impacts of the salmon subsidy on many coastal bear populations and the extent to which these populations transport marine‐derived nutrients into terrestrial food webs remains limited. Here, using a noninvasive genetic sampling approach capable of discriminating individuals, we show that bears in southwestern Alaska congregate in large numbers during the summer along small sockeye salmon spawning streams where direct observation is difficult. Affirming our first two predictions, bears moved freely among proximate streams within putative foraging neighborhoods but rarely moved between these neighborhoods either during or across years. Contrary to our third prediction, we did not observe consistent differences in bear detection rates among our focal streams despite considerable variation in observed average salmon run size (Quinn et al., 2017). Finally, we did not observe significant differences in estimated abundance and detection rates between female and male bears despite our expectation that the impacts of humans in our system might disproportionately deter male bears and lead to lower detectability. Our results shed new light on the importance of small salmon spawning streams to brown bears along the North Pacific Rim, reveal that potential for bears to mitigate the cost of variation in prey abundance by simultaneously foraging on salmon in multiple streams, suggest that stream neighborhoods may spatially structure coastal brown bear populations, and illustrate the utility of noninvasive genetic monitoring of bear individuals and populations as a methodological compliment to direct observation, camera trapping, and radio telemetry.

Daily brown bear foraging aggregations at waterfalls on the Brooks and McNeil rivers in Alaska can include as many as 60 (Smith, Herrero, & DeBruyn, 2005) and 74 (Gill & Helfield, 2012) individuals, respectively. Furthermore, over 100 individuals have been detected in the span of a few days within a 10 km^2^ core area around McNeil Falls (Sellers & Aumiller, 1994). These abundance estimates underscore the potential for spawning salmon to influence bear distribution and subsidize terrestrial ecosystems with marine‐derived nutrients. Questions remain, however, about the extent to which these areas represent other less prominent regions of the North Pacific Rim where bears and Pacific salmon come into contact. Focusing on small streams that are more typical of the coastal habitats where brown bears exploit spawning salmon throughout their geographic range, we estimated as many as 41 individuals using a single stream over the course of the 6‐week spawning season, and that four streams in 2013 and five streams in 2014 and 2015 were visited by at least 10 individual bears. Our abundance estimates do not indicate how many bears visited streams on a single day and include both cubs and, presumably, at least some individuals from the areas surrounding the streams that spent little time using the streams themselves (Kendall et al., 2008). These abundance estimates are nevertheless impressive because all of our focal streams were small and support modest salmon runs averaging between 1,025 and 11,312 fish (Quinn et al., 2017) and we only sampled short (2 km) stretches of all streams. Accordingly, our findings reveal that, much like the famous viewing areas along the Brooks and McNeil rivers, small spawning streams where predation efficiency is highest (Gende et al., 2004; Quinn et al., 2017) can attract large numbers of bears and are likely important resources for many coastal bear populations, given the ubiquity of these salmon habitats and foraging efficiency displayed by bears in small streams. By implication, declines or losses of salmon runs in small streams have the potential to affect the dynamics of bear populations along the North Pacific Rim and disrupt substantial nutrient flows that connect aquatic and terrestrial food webs and should be considered in conservation planning.

Foragers exploiting spatially and temporally dynamic resources are expected to use multiple patches in order to minimize lost opportunity costs (Stephens & Krebs, 1986). In accord with this prediction, we found that each year individual bears frequently moved between streams within neighborhoods. Indeed, numerous bears (*n* = 20) were detected using neighboring streams over the course of 48‐hr intervals; we could not assess use of adjacent streams on the same day because streams were checked every other day. By implication, bears may use this tactic to mitigate the risk posed by interannual variation in salmon abundance in particular streams.

In general, animals are predicted to display site fidelity to the degree that spatiotemporal predictability of dispersed resources rewards local familiarity (Andersson, 1980; Wiens, 1976). Along the North Pacific Rim, salmon predictably return to the same spawning grounds at roughly the same time each year, offering bears an opportunity to benefit from the experience gained from reusing particular spawning locations. Furthermore, brown bear fidelity to highly visible foraging areas has been documented (Sellers & Aumiller, 1994), their foraging efficiency improves as a function of familiarity with site‐specific features of salmon spawning habitat (Gill & Helfield, 2012), and avoidance of socially dominant conspecifics (Gende & Quinn, 2004) would presumably also be facilitated by local experience. Hence, we predicted that bears would tend to stay within the same neighborhood in a given year and return to the same foraging neighborhood year after year, rather than switch neighborhoods. In accord with this expectation, we documented frequent movement among streams within neighborhoods in a given year but little movement between neighborhoods. Although we cannot ascertain the mechanism underlying this pattern, the infrequency of movement between neighborhoods is consistent with the energy that would be required to traverse the lake (a swim of roughly 4 km) or move around it (a trip of roughly 11 km, Quinn et al., 2014). Moreover, among the bears that we recaptured across years, very few switched neighborhoods. This latter finding could reflect bears using geographic landmarks such as lakes to delineate space use boundaries during the salmon spawning season (Mesterton‐Gibbons & Adams, 2003). However, this result is unlikely to derive solely from the presence of a geographic barrier given the high mobility of brown bears (e.g., summer home ranges in Alaska can exceed 100 km^2^ [Barnes, 1990] and large travel distances (10 s of km) by bears targeting salmon [Glenn & Miller, 1980]). Moreover, we did document interneighborhood movement during a spawning season. Another possible explanation for this result is fitness costs stemming from reduced familiarity with local site features and perhaps increased competition with conspecifics (Gende & Quinn, 2004; Gill & Helfield, 2012), although our data do not allow for a test of this hypothesis. In sum, then, we determined movement between the two neighborhoods both during and across years to be possible but rare. To our knowledge, this study is the first to document individual fidelity to salmon foraging habitat where salmon spawn in small streams and bears are relatively cryptic (i.e., do not congregate at obvious migratory bottlenecks and are often obscured by brush). Loss of key prey populations that are the basis of such site fidelity has been cited as a conservation concern for other predators (e.g., fish‐eating killer whales (*Orcinus orca*) preying on Chinook salmon (*Oncorhynchus tshawytscha*), Hanson et al., 2010; gray wolves (*Canis lupus*) targeting marine resources; Darimont, Paquet, & Reimchen, 2009). By the same token, our results imply that loss of certain salmon runs, even if they are relatively small, could have disproportionately negative consequences for certain bears. Accordingly, there remains need for studies addressing the spatiotemporal scope and long‐term persistence of the neighborhood fidelity we have documented as well as its consequences for bear population demography.

In mammals, females tend to display greater site fidelity than males (Greenwood, 1980), and subadult male brown bears disperse farther than females and adult males (Manchi & Swenson, 2005). Our findings are generally consistent with this pattern; namely females were more likely than males to be recaptured in the same stream neighborhood across years and to be recaptured in at least three of the 4 years during which recapture was possible. In both cases, however, the sex differences were not significant relative to expectations based on the overall sex ratio of detected individuals, so confirmation of this tendency will require additional data. Nevertheless, persistent stream neighborhood use in brown bears may be female‐biased, and consequently bear population structure in coastal regions where Pacific salmon spawn may be driven largely by female philopatry. If female bears bequeath their space use patterns to their offspring, then our findings also raise the interesting possibility that spatial associations between brown bears and spawning streams could be behavioral traditions that are passed predominantly from mothers to female offspring (Morehouse, Graves, Mikle, & Boyce, 2016; Nielsen, Shafer, Boyce, & Stenhouse, 2013).

Brown bears are able to prolong their access to salmon by sequentially targeting streams with different run times (Deacy et al., 2016) Our results suggest that, instead of stemming from fidelity to particular streams, population structure in brown bears could arise from individuals consistently exploiting the same series of streams (or stream neighborhoods) each year. Accordingly, there is need for investigations that either track individual bears as they move among streams across multiple years or use noninvasive approaches to monitor interannual variation in detection patterns characterizing sequences of streams (or stream neighborhoods) across large landscapes.

The efficacy of our noninvasive genetic sampling approach depended on bears contacting the barbed wires while in the focal streams, so we expected that higher salmon availability might coax bears to spend more time foraging in certain streams (Hilderbrand, Hanley, et al., 1999, 1999), leading in turn to higher detection probability. Hence, given that our study streams vary with respect to average salmon run size (Quinn et al., 2017), we predicted that bear detection rates might differ across streams, with streams offering the most salmon also featuring the highest detection rates. None of the yearly Huggins closed‐capture models included the effect of stream identity, however, indicating that detection rates did not differ among the streams. It is possible that our analysis lacked the temporal scope and/or failed to capture sufficient variation in salmon abundance to rigorously test for differences in detectability among the sampled streams. Alternatively, our findings could indicate that any differences in residency patterns across the study streams either were negligible or did not affect detection rates. Future studies coupling noninvasive genetic population estimation with deployment of GPS collars to assess stream residency patterns (e.g., Flynn, Lewis, Beier, & Pendleton, 2007) are needed to address these latter scenarios.

Female brown bears are thought to be less sensitive to human presence than males, and may even use humans as shields against infanticidal males (Rode et al., 2006b; Steyaert et al., 2013, 2016). Because the bears in our study system are exposed to researchers, hunters, and residents of the nearby town of Aleknagik, we expected to find elevated female abundance and detectability relative to males. The overall pattern of abundance we observed was consistent with this expectation, namely female abundance was higher than that for males in all 3 years for which comparisons were possible on four streams and in 14 of the total of 18 possible comparisons across all of the streams. In all 14 of these cases, however, the 95% CI for females and males overlapped, indicating that the differences were not significant. Furthermore, the variable for sex‐specific detectability was only included in the top bear abundance model for 2015, and even in this case, the top model was not markedly better than the null model and the estimates for female and male detectability had overlapping 95% CI. Therefore, we conclude that, despite qualitative evidence for higher female abundance in all years and detection in 2015, our results do not demonstrate either increased female abundance or detectability on our focal streams. By implication, differences between female and male abundance in our system may have been too modest to discriminate using our noninvasive genetic CMR approach and to generate divergent stream residency and detection patterns. We also note, however, that inclusion of cubs may have hindered our ability to detect disparities in sex‐specific abundance and detectability, and that we lacked reference streams experiencing lower human activity with which to compare our findings. Accordingly, further analyses in our system that account for the age of detected bears and/or include streams with a reduced human footprint are warranted.

Interestingly, the four streams for which female abundance was consistently elevated, albeit qualitatively, were the four with the largest average sockeye salmon runs (Happy, Hansen, Bear, Yako; Quinn et al., 2017). Conversely, no such pattern existed on the two streams with relatively weak salmon runs (Eagle, Whitefish). Socially dominant bears displace subordinate individuals from areas of high salmon density (Elfström, Zedrosser, StØen, & Swenson, 2014; Gende & Quinn, 2004). Thus, it is possible that Eagle and Whitefish creeks are used disproportionately by socially subordinate bears, which are less sensitive to human presence irrespective of sex (Elfström et al., 2014), and consequently that abundance on these streams is less likely to be female biased. Studies capable of assessing age and social status would be necessary to evaluate this possibility.

There is increasing recognition that noninvasive sampling methods, including hair snagging for genetic analysis and camera trapping, can serve as safe and cost‐effective means of studying the behavior of large carnivores (Long et al., 2008). To date, most studies of bear abundance and behavior in salmon spawning habitat have relied on direct observation (e.g., Gende et al., 2004; Gill & Helfield, 2012) or tracking technology such as GPS collars (e.g., Deacy et al., 2016; Hilderbrand, Hanley, et al., 1999; Seryodkin et al., 2017), whereas noninvasive hair snaring has typically been used to explore patterns of bear distribution and abundance across larger landscapes (e.g., Apps, McLellan, Woods, & Proctor, 2004; Boulanger et al., 2008; Crupi, Waite, Flynn, & Beier, 2017). Here, we deployed unbaited hair snares at a much smaller scale to generate highly localized and sex‐specific estimates of brown bear abundance and detectability in two foraging neighborhoods, each consisting of a trio of streams. Several factors likely contributed to the success of our effort. Namely, we were able to deploy our barbed wires in a manner that took advantage of the linear movements brown bears are known to make up and down small spawning streams (Gende et al., 2004), sample over an ecologically appropriate time scale (the sockeye salmon spawning season; Boulanger et al., 2008; De Barba et al., 2010), and work along streams where bears tend to be more detectable (Rovang, Nielsen, & Stenhouse, 2015), especially because in our case the streams contained a natural attractant (the salmon). Our noninvasive approach is not suitable for exploring processes, such as continuous movement and fine‐scale resource selection, that would require higher‐resolution GPS telemetry data (Hebblewhite & Haydon, 2010). Rather, we suggest that it could be applied to explore small‐scale patterns of abundance, detection, and even movements in other systems that similarly lend themselves to unbaited hair snaring. For example, bear hair could easily be sampled and used for both CMR population estimation and individual monitoring where trail networks facilitate deployment of unbaited, single‐catch snares (Beier et al., 2005). Localized and noninvasive efforts of this nature (see Wheat, Allen, Miller, Wilmers, & Levi, 2016 for another approach using environmental DNA) are likely to be especially useful in cases where bears are difficult to observe directly and deployment of enough GPS collars to make inferences at the population level is intractable.

In summary, we used a noninvasive genetic approach to demonstrate not only that large numbers of brown bears congregate in small stream neighborhoods to exploit spawning Pacific salmon but also the utility of hair‐sampling wires for monitoring patterns of bear abundance and behavior at relatively fine spatial and temporal scales. Our results further underscore the importance of salmon to bears, as well as the potential for bears to act as vectors of consumer‐mediated nutrient transport, by highlighting the attractiveness of small streams as foraging patches. Given the site fidelity we documented, our results also suggest that stream neighborhoods, or perhaps series of neighborhoods characterized by sequential spawning intervals (Deacy et al., 2016), may be drivers of bear population structure along the North Pacific Rim. If such structure is commonplace, then the possibility that threats to local salmon runs may affect certain bears disproportionately will need to be weighed as part of management considerations. We acknowledge, however, the limited spatial and temporal scope of our investigation and, consequently, advocate for longitudinal and more spatially expansive inquiries addressing the broader applicability of our findings.

## CONFLICT OF INTEREST

None declared.

## AUTHOR CONTRIBUTIONS

All authors contributed substantially to the design and/or data analysis and interpretation of the work. Specifically, AW, TQ, CC, and LW contributed to the design of the study; AW, TQ, and CC conducted field sampling; AW, JA, AC, and LW were involved in data analysis; and all authors assisted with interpretation. All authors also contributed extensively to the writing and revision of the manuscript, and all have read and approved the present version. Finally, all authors have agreed to be accountable for all aspects of the work.

## DATA ACCESSIBILITY

Data available from the Dryad Digital Repository: https://doi.org/10.5061/dryad.1h8sr94.
