# Supplementary material for: Alaskan brown bears (Ursus arctos) aggregate and display fidelity to foraging neighborhoods while preying on Pacific salmon along small streams
Source: Ecol Evol. 2018 Aug 19;8(17):9048–61. doi: 10.1002/ece3.4431 (PMC6157690; doi:10.1002/ece3.4431)
Supplement: Supplementary file 1 [file ECE3-8-9048-s001.docx]

**Supporting information**

**Table S1**. Numbers of brown bear (*Ursus arctos*) hair samples collected (*N*) and analyzed (A) as part of a study using hair sampling barbed wires deployed along six sockeye salmon (*Oncorhynchus nerka*) spawning streams flowing into Lake Aleknagik (Wood River System, Alaska, USA) over the course of four summers (2012-2015). Streams are designated as comprising either the trio along the northern (N) or southern (S) shore of the lake.

|  | **2012** | | **2013** | | **2014** | | **2015** | |
| --- | --- | --- | --- | --- | --- | --- | --- | --- |
| **Stream** | Collected | Analyzed | Collected | Analyzed | Collected | Analyzed | Collected | Analyzed |
| Happy (N) | 12 | 10 | 123 | 71 | 8 | 6 | 164 | 54 |
| Hansen (N) | 33 | 16 | 132 | 83 | 58 | 23 | 271 | 75 |
| Eagle (N) | -- | -- | 40 | 26 | 134 | 49 | 201 | 63 |
| Bear (S) | 29 | 15 | 28 | 23 | 57 | 27 | 268 | 82 |
| Yako (S) | -- | -- | 68 | 46 | 44 | 26 | 256 | 72 |
| Whitefish (S) | -- | -- | 17 | 14 | 33 | 24 | 50 | 24 |
| **Totals** | 74 | 41 | 408 | 263 | 334 | 155 | 1,210 | 370 |

**Table S2.** Naïve (minimum number known to be alive; i.e., detected) and non-invasive genetic capture-mark-recapture (CMR) estimates of female and male brown bear abundance along six sockeye salmon spawning streams that flow into Lake Aleknagik in Bristol Bay, Alaska. Streams are grouped into those along the northern (N) and southern (S) shores of the lake. Estimates were generated using multi-session Huggins closed-capture models based on encounter histories for each stream over three summers (2013-2015) during which hair samples were collected with barbed wire for six weeks. CMR estimates are from the top model in each year and are accompanied by 95% confidence intervals (upper and lower bounds).

|  | Stream | Naïve estimate | CMR Estimate | Lower Confidence Limit | Upper Confidence Limit |
| --- | --- | --- | --- | --- | --- |
| **2013** | Happy (N) - females | 9 | 14.361 | 10.726 | 25.648 |
|  | Happy (N) - males | 3 | 4.787 | 3.351 | 12.095 |
|  | Hansen (N) - females | 11 | 17.550 | 13.260 | 29.986 |
|  | Hansen (N) - males | 8 | 12.765 | 9.469 | 23.459 |
|  | Eagle (N) - females | 4 | 6.383 | 4.542 | 14.470 |
|  | Eagle (N) - males | 5 | 7.978 | 5.754 | 16.771 |
|  | Bear (S) - females | 4 | 6.383 | 4.542 | 14.470 |
|  | Bear (S) - males | 1 | 1.596 | 1.062 | 6.707 |
|  | Yako (S) - females | 8 | 12.654 | 9.469 | 23.459 |
|  | Yako (S) - males | 5 | 7.978 | 5.754 | 16.771 |
|  | Whitefish (S) - females | 3 | 4.787 | 3.351 | 12.095 |
|  | Whitefish (S) - males | 3 | 4.787 | 3.351 | 12.095 |
| **2014** | Happy (N) - females | 2 | 4.098 | 2.382 | 13.523 |
|  | Happy (N) - males | 0 | 0 | -- | -- |
|  | Hansen (N) - females | 5 | 10.245 | 6.444 | 24.054 |
|  | Hansen (N) - males | 4 | 8.196 | 5.057 | 20.652 |
|  | Eagle (N) - females | 4 | 24.588 | 16.518 | 47.071 |
|  | Eagle (N) - males | 4 | 8.196 | 5.057 | 20.652 |
|  | Bear (S) - females | 9 | 18.436 | 12.147 | 37.288 |
|  | Bear (S) - males | 2 | 4.098 | 2.382 | 13.523 |
|  | Yako (S) - females | 6 | 12.294 | 7.851 | 27.406 |
|  | Yako (S) - males | 2 | 4.098 | 2.382 | 13.523 |
|  | Whitefish (S) - females | 4 | 8.196 | 5.057 | 20.652 |
|  | Whitefish (S) - males | 1 | 2.049 | 1.130 | 9.449 |
| **2015** | Happy (N) - females | 10 | 13.560 | 11.038 | 22.239 |
|  | Happy (N) - males | 6 | 11.824 | 7.626 | 26.858 |
|  | Hansen (N) - females | 14 | 18.988 | 15.645 | 29.127 |
|  | Hansen (N) - males | 8 | 15.765 | 10.370 | 33.446 |
|  | Eagle (N) - females | 11 | 14.919 | 12.184 | 23.971 |
|  | Eagle (N) - males | 13 | 25.618 | 17.339 | 49.698 |
|  | Bear (S) - females | 9 | 12.207 | 9.894 | 20.498 |
|  | Bear (S) - males | 5 | 9.853 | 6.271 | 23.534 |
|  | Yako (S) - females | 8 | 10.850 | 8.756 | 18.746 |
|  | Yako (S) - males | 2 | 3.941 | 2.340 | 13.088 |
|  | Whitefish (S) - females | 2 | 2.713 | 2.091 | 7.575 |
|  | Whitefish (S) - males | 3 | 5.912 | 3.619 | 16.692 |
